# Time Exposure Period of Bovine Oocytes to Sperm in Relation to Embryo Development Rate and Quality

**DOI:** 10.5402/2011/257627

**Published:** 2011-02-24

**Authors:** Marco Berland, Mario Frei, Oscar Peralta, Marcelo Ratto

**Affiliations:** ^1^Laboratory of Animal Reproduction, Veterinary School, Faculty of Natural Resources, Catholic University of Temuco, P.O. Box 4780000, Temuco, Chile; ^2^Department of Animal Sciences, Faculty of Veterinary Sciences, Southern University of Chile, P.O. Box 5090000, Valdivia, Chile

## Abstract

The objective of the study was to determine the effect of different bovine gamete coincubation times on fertilization and embryo development performance. *In vitro* matured COCs were co-incubated with sperm at a concentration of 1.5 × 10^6^ spermatozoa/ml in TALP medium for 3 hours (T 3, *n* = 362), 6 hours (T 6, *n* = 358), or 18 hours (T 18, *n* = 350). At the end of the coincubation period COCs from times 3 and 6 groups were post-incubated in a new well of fertilization medium without sperm for additional 15 and 12 h, respectively. Cumulus Oocyte Complexes from the T 18 were co-incubated with the sperm suspension for 18 hours. Presumptive zygotes were cultured for 9 days and embryo development was evaluated on days 2, 8, and 9. Thirty blastocysts from each group were stained and total number of nuclei was recorded. The mean (± SEM) percentages of zygotes to develop into ≥2 cell stage were 71.9 ± 5.0; 72.5 ± 5.3 and 81.2 ± 6.1 % for T 3, 6, and 18, respectively, on day 2 and they did not differ (*P* = .3) among groups. The mean percentage of blastocysts developed on day 8 (25.6 ± 2.8; 24.2 ± 3.3; 28.4 ± 4.2 % for T 3, 6, and 18, resp.) did not differ (*P* = .4) among groups. The total number of embryonic nuclei was greater (*P* < .05) for the blastocysts produced from the shortest co-incubation time (T 3).

## 1. Introduction

The time of gamete coincubation play an important role in the process of bovine *in vitro* fertilization. Previously, it has been reported that bovine cumulus oocyte complexes (COCs) co-incubated for 18–24 hours with sperm at a concentration of 1-1.5 × 10^6^ per mL achieve acceptable rates of *in vitro* fertilization and embryo development [1–3]. However, it has also been documented [1], that reducing gamete coincubation to 10 hours using the same sperm concentration achieve similar rates of *in vitro* fertilization and embryo development. Although, the prolonged coincubation time (18–24 h) has been shortened by significantly increasing sperm concentration up to 6 × 10^6^/mL [4, 5], it is plausible that both high sperm concentration or prolonged interval of gamete coincubation may result in an excess of dead sperm which may induce zona pellucida hardening and compromise *in vitro *fertilization and embryo viability [6–8]. Indeed, increased number of dead sperm has been reported to induce high levels of reactive oxygen species (ROS) production and hydrolytic enzymes that may affect oocyte viability and subsequent embryo developmental capacity [2, 4, 9].

Alternative protocols have been proposed to avoid prolonged exposure of the oocyte to the high sperm concentration during *in vitro* fertilization. For instance, denuded pig oocytes have been co-incubated *in vitro* with sperm for 10 minutes and then transferred into fresh *in vitro* fertilization medium without sperm for additional 5-hour culture [10]. Although, the use of this protocol has been showen to increase blastocyst formation [10], other pig studies reported that the efficiency of *in vitro* embryo production was not improved by this method [11, 12]. Apparently the increase in blastocyst development was associated with a male effect and sperm concentration instead with the period of gamete coincubation. 

A previous report in cattle [13] has documented that COCs co-incubated with sperm for 1.5–2 h followed by a post-incubation in sperm-free fertilization medium did not affect blastocyst development (25.5%) when compared to an 18–20-hour coincubation control (24.5%). Despite these results and others [1, 13], the reason for using prolonged 18–20-hour gamete coincubation interval has not been scientifically supported and may only be the consequence of simplified protocols that allow an overnight incubation.

Evidence reported in cattle [1, 14] suggest that gamete coincubation for 5-6 h results in lower rates of *in vitro* fertilization and embryo development, but higher quality embryo. Moreover in humans [6, 7, 15], shortening gamete coincubation period to 1 h resulted in increased proportion of blastocyst formation and implantation rate following embryo transfer. These studies suggest that shortening gamete coincubation may reduce damage associated to prolonged exposure of embryos to dead sperm and may result in higher quality embryos. 

Here we proposed to determine the effect of different bovine gamete coincubation times on *in vitro* embryo development rates and quality.

## 2. Material and Methods

### 2.1. Oocyte Collection and In Vitro Maturation

Ovaries were collected from a local abattoir and transported to the laboratory in 0.85% saline supplemented with 100 mg/ml of Streptomycin and 80 mg/mL Sodium Penicillin G at a temperature of 35–38°C within 3 h of collection. Cumulus Oocyte Complexes were obtained by aspiration of 3–6 mm in diameter follicles from the ovarian surface with a 19 G needle attached to a 10 mL syringe. Follicular fluid content was transferred to a 90-mm plastic petri dish (Falcon), and COCs were localized and classified under stereomicroscope at a magnification of 40x. COCs were washed twice in Phosphate Buffer Saline (D-PBS, Gibco, Grand Island, NY, USA) supplemented with 0.3% of Bovine Serum Albumin (BSA Fraction V, Sigma-Aldrich, St Louis, MO, USA). Groups of 50 COCs were *in vitro* cultured in four-well dishes (Nunc, Roskilde, Denmark) with 500 *μ*L of Tissue Culture Medium 199 (TCM-199, Sigma) supplemented with 25 mM HEPES, 10% Calf Serum (Gibco), 0.2 Mm Sodium Pyruvate (Sigma), 40 *μ*g/mL of FSH (Folltropin-V, Bioniche, Belleville, ON, Canada), 5 *μ*g/mL of LH (Lutropin-V, Bioniche) and 1 *μ*g/mL of Estradiol (Sigma) for 24 hrs at 39°C, 5% CO_2_ and high humidity. After *in vitro* culture, COCs were washed twice in fertilization medium TALP (Thyroid Albumin Lactate Pyruvate) [16] supplemented with 10 *μ*g/mL of heparin, 6 mg/mL BSA (Fatty Acid-Free, Sigma) and 0.2 mM Sodium Pyruvate, and 5 *μ*g/mL of gentamicin.

### 2.2. Sperm Preparation and In Vitro Fertilization

Spermatozoa were obtained from frozen-thawed semen collected from one fertile bull (ABS, American Breeders Service, DeForest, WI, USA). Thawed sperm were washed in a discontinuous gradient of 45/90% Percoll (Sigma) using centrifugation at 700 g for 20 min. The pellet was resuspended with washing medium TALP containing 6 mg/mL BSA (Fraction V, Sigma), 1.0 mM Sodium Pyruvate and 5 *μ*g/mL of gentamicin and centrifuged once again at 250 g for 5 minutes. The final pellet was resuspended with *in vitro *fertilization medium at a concentration of 1.5 × 10^6^ spermatozoa/mL. Fifty COCs were transferred to 500 *μ*L of sperm suspension in four-well dishes.

### 2.3. Gamete Coincubation

Cumulus Oocyte Complexes were co-incubated with sperm for 3 h (3 h incubation time, [T3], *n* = 362), 6 hours (6 h incubation time, [T6], *n* = 358) or 18 hours (18 h incubation time, [T18], *n* = 350). After coincubation, COCs with sperm attached to their cumulus from the 3- and 6-hour incubation times were removed from their wells, washed and postincubated in new wells of fertilization medium without sperm for additional 15 and 12 h, respectively. Cumulus Oocyte Complexes for the 18-hour incubation time were not removed from their original drops and they were co-incubated with the original sperm concentration for 18 h according to the interval established for the basic bovine IVF protocol.

### 2.4. In Vitro Culture

After *in vitro* fertilization, COCs from all time intervals (3, 6, or 18 h incubation times) were cocultured with bovine oviductal epithelial cells (BOECs) as previously described [17]. Briefly, oviducts collected from abattoir were trimmed free of connective tissue and washed in PBS. The luminal tissue was harvested by scraping the oviducts on the outside with a glass slide. The content was washed twice with 10 mL of TCM-199 supplemented with 2% of Calf Serum (CS), 0.2 mM Sodium Pyruvate and then cultured in a tissue culture flask with 10 mL of M-199 supplemented with 10% CS for 48 h at 39°C, 5% CO_2_, and high humidity. Presumptive zygotes were transferred to 50 *μ*L culture drops of M-199 supplemented with 1 *μ*L of BOEC and cultured for 9 days at 39°C, 5% CO_2_ and high humidity.

### 2.5. Embryo Development and Total Cell Number of Blastocysts

Early cleavage was evaluated on day 2 after *in vitro* fertilization (Day 0 = *in vitro* insemination) and blastocyst formation and hatched blastocysts were recorded on days 8 and 9 of *in vitro* culture, respectively. Blastocysts from days 8 of *in vitro* culture (*n* = 30/per group) were stained with bisbenzimide (Bis, Hoechst 33242, Sigma) and the total number of embryonic nuclei were counted using fluorescence microscopy.

### 2.6. Statistical Analysis

Data from embryo development were *arcsin*-transformed and then analyzed using one-way analysis of variance (ANOVA). Total number of embryonic nuclei was analyzed using one-way ANOVA. Where significant differences were determined, mean values were analyzed by Tukey's post hoc test using STATISTICA (Copyright@ Stat Soft Inc, 2003).

## 3. Results

Nine hundred and eighty presumptive zygotes (*n* = 6 replicates) cocultured on bovine oviductal epithelial cells were evaluated for embryo development, while 90 presumptive zygotes (*n* = 3 replicates) cultured under the same conditions were submitted for fluorescence staining to count embryonic nuclei. 

The mean (±SEM) percentages of bovine COCs to develop to the 2-3, 4–8, and >8 cells stage on day 2 after *in vitro* fertilization did not differ (*P* = .3) among different intervals of gamete coincubation (Table 1). Although, the mean percentage of blastocysts developed on day 8 and hatched blastocysts observed on day 9 of *in vitro *culture did not differ (*P* = .4) among different coincubation intervals; the total number of embryonic nuclei was highest (*P* < .05) for the T3 interval compared to those observed in the remaining groups (Table 2).

## 4. Discussion

Shortened gamete coincubation intervals of 3 or 6 h followed by a postincubation in fertilization medium without sperm resulted in similar rates of embryo development compared with a prolonged interval of 18 h. These results suggest that sperm can interact and attach to the cumulus cells in a brief period of time (3 or 6 h) and that further incubation in medium without sperm has no effect on fertilization and embryo development. Indeed, the shortest interval increased the quality of blastocysts developed on day 8 as evidenced by the significantly higher total mean number of embryonic nuclei observed. Early attempts to shorten gamete coincubation intervals in the bovine have been showed to decrease embryo cleavage [1, 14, 18]; however, it has been documented that a high proportion of blastocysts are developed from the total embryos cleaved [14]. 

Our data suggest that prolonged gamete coincubation may have a deleterious effect on subsequent embryo development. As mentioned before, it is plausible that prolonged interval of gamete coincubation may result in an excess of dead sperm which may induce zona pellucida hardening and compromise *in vitro *fertilization and embryo viability [6–8]. Recent studies demonstrate that ROS levels in zygotes and ROS generated by spermatozoa are significantly higher after increase at 4 and 18 h after fertilization, respectively [9, 19]. The negative effect of ROS production during fertilization and embryo development has been extensively documented (e.g., reviews by [20, 21]). Furthermore, presence of antioxidants including *β*-Mercaptoethanol and Vitamin E has been showen to regulate concentration of ROS and increase the rate of development of embryos to the blastocysts stage [22, 23]. Alternatively, some studies have suggested that prolonged gamete coincubation (16 h) results may affect embryo development by increasing rates of polyspermy [7]. In spite of, the potential risk of polyspermy in prolonged exposure to spermatozoa is high, further research is required to address this effect. A recent bovine study [4] demonstrated that a prolonged gamete coincubation interval (6 to 18 h) using Bracket-Oliphant as fertilization medium decreased the rate of blastocyst formation. However, the situation can be reversed when the fertilization medium is replaced for more complex media such as TCM-199 or KSOM for which the prolonged gamete coincubation apparently does not affect the rate of blastocysts development [4]. In our study, TALP medium showed no adverse affects over embryo development rates after 18 h of incubation. 

The rate of blastocyst development, from the 3- and 6-hour coincubation intervals, respectively, observed in this study was similar to 25.5% reported in a preliminary bovine study using a similar protocol [13] except that the gamete coincubation interval was shorter (1.5–2 h). However, the authors in this study did not report the rate of hatched blastocysts and total number of embryonic nuclei. Although we could not find significant differences in blastocyst development and hatched blastocysts among coincubation intervals, the rate of hatched blastocysts for all the intervals was lower than previously reported in other bovine studies IVF [4, 24]. 

Based on the results of our study, we conclude that shortening gamete coincubation intervals to 3 or 6 h followed by a incubation in fertilization medium without sperm, resulted in similar rates of embryo development compared with the prolonged (18 h) interval commonly used for bovine IVF protocols. The data suggest that shorter incubation times increased blastocyst quality at day 8 of development as evidenced by the presence of a greater total mean number of embryonic nuclei. Further experiments are needed to confirm this effect.

## Figures and Tables

**Table 1:** Effect of different gamete coincubation intervals on bovine embryo development on day 2 after *in vitro* fertilization (mean ± SEM).

Coincubation interval (h)	Oocytes (*n*)	Embryo development (%)*			
		2-3 cells	4–8 cells	>8 cells	Total
T 3	362	11.4 ± 5.6	47.8 ± 6.5	12.6 ± 4.7	71.9 ± 5.0
T 6	358	7.6 ± 4.5	47.5 ± 7.1	16.7 ± 5.3	72.5 ± 5.3
T 18	350	10.3 ± 5.2	53.7 ± 5.9	17.1 ± 5.9	81.2 ± 6.1

*The proportion of embryo development was estimated by dividing the number of cleaved embryos at different stage by the total number of oocytes.

**Table 2:** Effect of different gamete coincubation intervals (mean ± SEM) on blastocysts development, hatched blastocysts, and total number of embryonic nuclei.

Coincubation interval (h)	Oocytes (*n*)	Blastocysts*Day 8 (%)	Hatched Blastocysts*Day 9 (%)	N*º* of embryonic nuclei (*n* = 30/per Group)
T 3	362	25.6 ± 2.8	23.4 ± 3.2	128.2 ± 4.5^a^
T 6	358	24.2 ± 3.3	24.0 ± 4.3	112.2 ± 6.1^b^
T 18	350	28.4 ± 4.2	25.9 ± 3.8	111.0 ± 5.7^b^

^a,b^Within columns, values with no common superscript are different (*P* < .05). *The proportion of blastocysts and hatched blastocysts were estimated by dividing the total number of blastocysts and hatched blastocysts by the total number of oocytes and blastocysts respectively among intervals of time.
